# Editorial: Immunity to Parasitic Infections in Pregnancy

**DOI:** 10.3389/fimmu.2021.813446

**Published:** 2021-12-16

**Authors:** Justin Y. A. Doritchamou, Elizabeth H. Aitken, Adrian J. F. Luty

**Affiliations:** ^1^ Laboratory of Malaria Immunology and Vaccinology, National Institute of Allergy and Infectious Diseases, National Institutes of Health, Bethesda, MD, United States; ^2^ Department of Infectious Diseases, The Peter Doherty Institute of Infection and Immunity, University of Melbourne, Parkville, VIC, Australia; ^3^ Department of Microbiology and Immunology, The Peter Doherty Institute of Infection and Immunity, University of Melbourne, Parkville, VIC, Australia; ^4^ Université de Paris, MERIT, IRD, Paris, France

**Keywords:** pregnancy, parasitic infections, immunity, therapeutics, vaccine, malaria, toxoplasmosis, trypanosomiasis

## Introduction

Despite having a degree of pre-existing immunity to plasmodial and other parasitic infections, pregnancy may render women more susceptible to these pathogens than their non-pregnant counterparts due, at least in part, to pregnancy-related alterations in immune responsiveness. Parasitic infections during pregnancy represent a major risk to maternal and fetal health (1–3). The maternal immune system must establish a balance between immune tolerance of the semi-allogeneic fetus and appropriate immune responses to pathogens. The particular case of infection with *Plasmodium falciparum*, the cause of the syndrome known as placental malaria (PM), illustrates the pathological consequences of excessive inflammatory responses on fetal development and maternal health. Not all parasitic pathogens are as virulent, but protozoa such as *Toxoplasma gondii* or *Trypanosoma cruzi* also challenge the maternal immune system and affect susceptibility to infections in early life. Thus, improved understanding of host-parasite interactions and the underlying immune mechanisms involved in parasitic infections during pregnancy will help define more efficient and effective approaches to tackle the burden of these infections and improve maternal-fetal health.

*Plasmodium* and other human parasitic species rely largely on their well-developed ability to manipulate the immune system to their own advantage, allowing them to establish chronic infections. On the other hand, pregnant women can develop a protective immunity that relies on both humoral and cellular immune mechanisms. The six original research and six review articles, published in this Research Topic, provide insights into our understanding of the diverse mechanisms involved in generating immune response to toxoplasmosis, trypanosomiasis and plasmodial infections, in the context of pregnancy, their impact on pregnancy outcomes, and disease control strategies including vaccines.

## Toxoplasmosis and Trypanosomiasis During Pregnancy

Chagas disease and toxoplasmosis, caused by *Trypanosoma cruzi* and *Toxoplasma gondii* (respectively), are two parasitic infections congenitally transmissible to the child, causing perinatal morbidity and mortality (2, 3). Medina et al. demonstrate these parasites induce a differential microRNA profile in human placental explants in an *ex-vivo* model. Furthermore*, in silico* analysis of the differentially expressed miRNAs reveals the predicted-targets regulated different cellular processes involved in cell development and immunity. Barros et al. review current advances and perspectives in the development of vaccines in congenital toxoplasmosis, highlighting the need for investigations to study the nature of protective immunity at the maternal-fetal interface; there has been no consensus on the optimal immune response required to protect both mother and fetus in congenital infection using live or subunit vaccination approaches.

## Malaria During Pregnancy: Pathogenesis

In malaria infection during pregnancy, there has been a strong interest to understand how malaria infection and placental pathology may result in adverse outcomes. Sarr et al. examine granulocytes and markers of neutrophil activation in pregnant women with, and without, *P. falciparum* and HIV infections. They note that granulocyte counts in peripheral and placental blood are differentially affected by infection and that markers of activation are increased in those with PM. Kabyemela et al. investigate potential causes of fetal anemia in a region of high malaria transmission, and report that fetal anemia is not associated with the presence of *P. falciparum* infection at delivery; they identify other factors (including cytokines, α thalassemia, and soluble transferrin receptor), which have been previously implicated in the pathogenesis of anemia. Finally, Chua et al. review our current knowledge on the different pathological pathways leading to low birthweight of babies from mothers that experienced malaria during pregnancy. Current strategies to prevent and manage malaria in pregnancy as well as potential therapeutic interventions that may improve birth outcomes are also discussed.

## Malaria During Pregnancy: Immunity

Although common in pregnancy, maternal anemia can be exacerbated by malaria resulting in increased risk of maternal morbidity and mortality (1). Wiebe and Yanow review the possible roles played by antibodies to malarial variant surface antigens (VSA) on infected erythrocytes (IEs) in protecting mothers from maternal anemia, and discuss current knowledge gaps. It appears that pathways leading to anemia in PM are complicated and multifactorial, with several conflicting studies finding no-, mixed- or inverse-associations between anti-VSA antibodies and maternal anemia during pregnancy malaria.

Immune competence in the infant during malaria infection is also reviewed by Callaway et al. in order to translate our current understanding of fetal and neonatal immunology into safe and immunogenic vaccines that can be administered in early infancy. The authors highlight that the fetus is immunologically competent and can mount adaptive B and T cell responses to perinatal pathogens *in utero*, providing proof-of-concept that induction of protective immunity prior to birth may be possible.

## VAR2CSA Antibodies and Placental Malaria Vaccine Development

*P. falciparum* parasites, which cause PM, express the protein VAR2CSA on the surface of IEs (4), and pregnant women with elevated levels of high avidity antibodies to VAR2CSA early in pregnancy had a reduced risk of PM at delivery (5). Vanda et al. demonstrate that high avidity antibodies to VAR2CSA are predominately restricted to the DBL5 domain of VAR2CSA, and their levels increase from first to second pregnancy (with affinity maturation of antibody taking place primarily during the second pregnancy), while positively influencing the baby’s birthweight. Elevated levels of VAR2CSA-specific total IgG and cytophilic IgG3 during pregnancy are also associated with higher birth weights, as shown by Tornyigah et al. The authors evaluate the subclass of anti-VAR2CSA IgG responses in a cohort of pregnant Beninese women using the PAMVAC candidate vaccine antigen, and demonstrate that cytophilic IgG1 and IgG3 responses to VAR2CSA are the most frequent, whilst high levels of IgG4 are associated with reduced risk of placental infections. This study provides evidence that protection induced by VAR2CSA antibodies results from coordinated activities between both cytophilic and non-cytophilic antibodies.

Consistent with reports from Vanda et al. and Tornyigah et al., McLean et al. demonstrate that levels of VAR2CSA IgG are significantly associated with increased birth weight in a longitudinal study of women infected with malaria during pregnancy. In particular, the authors show that women who had been infected by mid-pregnancy at enrolment had higher levels of antibodies to VAR2CSA associated with a reduced risk of adverse outcomes, in contrast to women uninfected at enrolment. Findings from this study highlight that high levels of VAR2CSA antibodies early in pregnancy may lead to better pregnancy outcomes.

VAR2CSA is the leading vaccine candidate to prevent PM particularly in first-time mothers who are at greater risk of poor pregnancy outcomes due to PM. Tomlinson et al. critically review the host defense evasion mechanisms employed by VAR2CSA-expressing *P. falciparum* during PM. The review focuses on VAR2CSA roles in PM pathogenesis, including cytoadhesion in the placenta as well as modulation of the placental microenvironment by pregnancy-specific IEs to escape recognition by protective antibodies.

After decades of preclinical development of PM vaccines, two VAR2CSA-based subunit vaccines have recently been tested in phase 1 trials (6, 7). In their review, Gamain et al. discuss recent advances in PM vaccine development, with a focus on recent clinical data, and outline the next clinical steps required to improve our understanding of vaccine-induced immunity and accelerate second generation of PM vaccine development.

## Conclusion

In summary, the burden of parasitic diseases in pregnant women remains a major global health problem. The published articles in this Research Topic show the complexity of the relationship between infection, immunity, and clinical outcomes of parasitic infections in pregnancy. Although *P. falciparum* is a dominant pathogen in parasitic infections during pregnancy, this collection also notes the roles of other protozoa that can challenge the maternal-fetal immunity. These articles also provide a better understanding of the diverse mechanisms involved in generating immune responses to parasitic infections during pregnancy and their impact on pregnancy outcomes that will inform future therapeutic approaches, including vaccination, to protect the mother and fetus health.

## Author Contributions

JD and EA wrote sections of the manuscript. All authors contributed to manuscript revision, read, and approved the submitted version.

## Funding

JD is supported by the Intramural Research Program of the National Institute of Allergy and Infectious Diseases, National Institutes of Health. EA is supported by a grant from the National Health and Medical Research Council of Australia (GNT1143946).

## Conflict of Interest

The authors declare that the research was conducted in the absence of any commercial or financial relationships that could be construed as a potential conflict of interest.

## Publisher’s Note

All claims expressed in this article are solely those of the authors and do not necessarily represent those of their affiliated organizations, or those of the publisher, the editors and the reviewers. Any product that may be evaluated in this article, or claim that may be made by its manufacturer, is not guaranteed or endorsed by the publisher.

## References

[1] Brabin BJ. An Analysis of Malaria in Pregnancy in Africa. Bull World Health Organ (1983) 61:1005. 6370484 PMC2536236

[2] Carlier Y Truyens C Deloron P Peyron F. Congenital Parasitic Infections: A Review. Acta Tropica (2012) 121:55–70. doi: 10.1016/j.actatropica.2011.10.018 22085916

[3] Błaszkowska J Góralska K. Parasites and Fungi as a Threat for Prenatal and Postnatal Human Development. Ann Parasitol (2014) 60:225–34. 25706418

[4] Salanti A Staalsoe T Lavstsen T Jensen ATR Sowa MPK Arnot DE. Selective Upregulation of a Single Distinctly Structured Var Gene in Chondroitin Sulphate A-Adhering Plasmodium Falciparum Involved in Pregnancy-Associated Malaria. Mol Microbiol (2003) 49:179–91. doi: 10.1046/j.1365-2958.2003.03570.x 12823820

[5] Tutterrow YL Salanti A Avril M Smith JD Pagano IS Ako S. High Avidity Antibodies to Full-Length VAR2CSA Correlate With Absence of Placental Malaria. PLoS One (2012) 7:e40049. doi: 10.1371/journal.pone.0040049 22761948 PMC3383675

[6] Mordmüller B Sulyok M Egger-Adam D Resende M de Jongh WA Jensen MH. First-In-Human, Randomized, Double-Blind Clinical Trial of Differentially Adjuvanted PAMVAC, A Vaccine Candidate to Prevent Pregnancy-Associated Malaria. Clin Infect Dis (2019) 69:1509–16. doi: 10.1093/cid/ciy1140 PMC6792113 30629148

[7] Sirima SB Richert L Chêne A Konate AT Campion C Dechavanne S. PRIMVAC Vaccine Adjuvanted With Alhydrogel or GLA-SE to Prevent Placental Malaria: A First-in-Human, Randomised, Double-Blind, Placebo-Controlled Study. Lancet Infect Dis (2020) 20:585–97. doi: 10.1016/S1473-3099(19)30739-X 32032566

